# Value of PET radiomic features for diagnosis and reccurence prediction of newly diagnosed oral squamous cell carcinoma

**DOI:** 10.1038/s41598-025-02305-3

**Published:** 2025-05-20

**Authors:** Elisabeth Pfaehler, Andreas Schindele, Alexander Dierks, Cornelius Busse, Joachim Brumberg, Alexander C. Kübler, Andreas K. Buck, Christian Linz, Constantin Lapa, Roman C. Brands, Olivia Kertels

**Affiliations:** 1 Nuclear Medicine, Faculty of Medicine, University of Augsburg, Augsburg, Germany; 2 Department of Oral and Maxillofacial Plastic Surgery, University Hospital of Würzburg, Würzburg, Germany; 3 Department of Nuclear Medicine, University Hospital of Freiburg, Freiburg, Germany; 4 Department of Nuclear Medicine, University Hospital of Würzburg, Würzburg, Germany; 5 Department of Oral and Maxillofacial Plastic Surgery, University Hospital of Cologne, Cologne, Germany; 6 Bavarian Cancer Research Center, Erlangen, Germany; 7 Institute of Diagnostic and Interventional Neuroradiology, Klinikum rechts der Isar, School of Medicine and Health, Technical University of Munich, Munich, Germany

**Keywords:** Radiomics, Squamous cell carcinoma of the oral cavity, Positron emission tomography, Prediction, Cancer imaging, Oral cancer

## Abstract

Oral Squamous Cell Carcinoma (OSCC) represents more than 90% of oral cancers. The usefulness of radiomic features extracted from PET images of OSCC patients to predict tumor characteristics such as primary tumor stage (T-stage), or tumor grade has not been investigated yet. In this prospective study, 112 patients with newly diagnosed, treatment-naïve OSCC were included. Tumor segmentation was performed using three strategies, the majority vote of these segmentations was used to calculate 445 radiomic features. Features instable over segmentation methods and features highly correlated with volume, SUV_max_, and SUV_mean_ were eliminated. A Random Forest classifier was trained to predict T-stage, tumor grade, lymph node involvement, and tumor recurrence. Stratified 10-fold cross-validation was performed. Evaluation metrics such as accuracy and area under the curve (AUC) were reported. SHAP dependence plots were generated to understand classifier decisions. The classifier reached a mean cross-validation AUC of 0.83 for predicting T-stage, an AUC of 0.56 for the grading of the primary tumor, a mean AUC of 0.64 for lymph node involvement, and a mean AUC of 0.63 for recurrence. In patients with newly-diagnosed OSCC, radiomics might have some potential to predict T-stage. These results need to be validated in a larger patient cohort.

**Supplementary Information:**

The online version contains supplementary material available at 10.1038/s41598-025-02305-3.

## Introduction

Cancer of the oral cavity and the oropharynx is the sixth most common tumor entity and the ninth most frequent cause of death worldwide. Oral squamous cell carcinoma (OSCC) accounts for more than 90% of all oral cancers with over 300,000 new cases each year^1^. Early detection and treatment of OSCC are critical for improving patient outcomes and survival rates^2^. However, the tumor diagnosis is determined late in up to 50% of patients. Thus, the condition is associated with a survival rate of approximately 60%, and an estimated recurrence rate of 30% at 5-year follow-up^3,4^. The presence of cervical lymph node metastases is one of the most important adverse prognostic factors^5–7^. Distant metastases, although rare, are generally considered incurable and therefore alter the therapeutic regimen^8^.

For newly diagnosed OSCC, precise initial tumor staging to determine the individual diagnosis, treatment, and prognosis is necessary. For classification, the extent of the primary tumor (T-stage), lymph node involvement (N-stage), and presence of distant metastasis (M-stage) are assessed. In addition, histopathologic evaluation of tumor grading is used to characterize biologic tumor differentiation.

The value of positron emission tomography (PET) using the tracer [^18^F]-fluorodeoxyglucose (FDG) in the preoperative staging of head and neck SCC has been demonstrated by various studies with a high accuracy for the detection of otherwise occult cervical nodal and distant metastases^9^. However, false negative results may be seen in very small lymph nodes or tumors of low metabolic activity and whether FDG-PET has the sensitivity to replace the conventional neck dissection is debated^10–13^.

Thus, it would be of great clinical value to determine an additional image biomarker extracted from the PET images that would give further information on tumor stage or particularly lymph node involvement. Additionally, since tumor recurrence is a well-established important prognostic factor^14^ portending a lower probability to survive the disease^15^, very early detection of cancer recurrence or prior identification of patients at risk is of high interest.

Tumor grade is a measure on tissue differentiation^16^. Hereby, a heterogeneous tumor indicates a higher tumor grade and more aggressive biologic behavior. This heterogeneity as well as other tumor characteristics could be identified by radiomic features^17^. Radiomic features are calculated from the segmented tumor and are describing tumor shape, intensity statistics, and texture. Therefore, radiomic features could provide additional information on the diagnosis and prognosis of OSCC patients.

Radiomic features showed promising results in a large number of studies^18,19^ for several cancer types. For OSCC patients, radiomic features of Computed Tomography (CT) and PET images outperformed a model using exclusively clinical parameters such as T-, N-, and overall stage for survival prediction^20^. In^21^, the authors selected three radiomic features extracted from PET images of OSCC patients to predict overall and progression-free survival. The model combining clinical parameters and radiomic features outperformed the model using clinical parameters only.

Despite encouraging results, radiomic features are yet not used in the clinic as their benefit is not entirely clear. Several studies demonstrated a high correlation to tumor volume for a large number of features^22,23^. Additionally, some studies demonstrated no benefit of radiomic features for different tumor entities^24,25^.

In this paper, we investigated if radiomic features extracted from PET images of patients suffering from newly diagnosed, treatment-naïve OSCC can be used to predict (a) primary tumor stage, (b) tumor grade, (c) lymph node involvement, and (d) recurrence. Hereby, we included only features that are (a) stable across segmentation algorithms and (b) not dependent on volume, maximum (SUV_max_), and mean (SUV_mean_) standardized uptake values (SUV). In order to give an explanation on model decisions, we analyzed SHapley Additive exPlanations (SHAP) values.

## Materials and methods

### Study population

This study is an additional analysis of a prospective study previously published^26^. The institutional review board approved this study, and written, informed consent was obtained from all participants (clinical trial number NCT04280159).

A total of 138 patients with clinical suspicion of OSCC were prospectively enrolled from June 1, 2013, to January 31, 2016. Whole-body [^18^F]FDG PET/CT was performed before further invasive interventions (panendoscopy and/or acquisition of biopsy samples). All patients underwent tumor surgery within two weeks of the imaging work-up. Resected primary tumors and lymph nodes were histopathologically evaluated. Histopathology served as standard of reference for the assessment of T-stage, tumor grade and metastatic lymph node involvement.

Patients included in this study had received no previous treatment. From the 138 patients, 13 yielded a cancer different from OSCC. Thirteen subjects did not demonstrate FDG uptake and were also excluded, leading to 112 patients in this analysis. Detailed inclusion and exclusion criteria can be found in^26^.

For the classification of recurrence, all patients with a follow-up of less than 12 months were excluded, leading to a total number of 78 patients that were included in the tumor recurrence sub-group.

### Dataset

All images were acquired on a PET/CT system (Siemens Biograph mCT 64; Siemens Healthineers, Erlangen, Germany) after fasting for 4 to 6 h. Prior to [^18^F]FDG injection (300 ± 25 megabecquerels), blood glucose levels were less than 160 mg/dL. PET scans started 60 min after injection for 2 min per bed position. Subsequently, transmission data were obtained using contrast-enhanced CT with 180 mAs and 120 kV. PET data were reconstructed iteratively with the vendor-provided ordered-subset expectation-maximization algorithm (3 iterations, 24 subsets, gaussian filtering of 2.0 mm full-width-at-half-maximum) with attenuation correction.

### Tumor segmentation

Primary tumors were segmented by one experienced nuclear medicine physician using three segmentation strategies:

- MAX41: All voxels with 41% or more of the maximum standardized uptake value (SUV_max_)^27^.
- SUV2: All voxels with a SUV $\:\ge\:$ 2^27,28^.
- SUV4: All voxels with a SUV $\:\ge\:$ 4^29^.

Based on these segmentations, a majority vote (MV) was determined which included all voxels marked by at least two of the mentioned segmentation methods^30,31^. Radiomic features were calculated from all segmentations to assess feature robustness to segmentation differences. Features extracted from the MV segmentations were used for classification as this method results in robust segmentations^32^.

### Calculation of radiomic features

445 radiomic features were calculated from the segmented Volume-Of-Interests using the RaCaT software version v.1.27^33^ which is in line with the Image Biomarker Standardization Initiative^34^. Images were resampled to isotropic voxels of 2 mm using tri-linear interpolation as recommended by *Pfaehler et al.*^35^. The calculated features include 6 standard PET metrics calculated before interpolation, 28 first-order, 24 shape, 129 grey-level co-occurrence matrix (GLCM), 96 grey-level run-length matrix (GLRLM) features, 48 grey-level size zone matrix (GLSZM), 15 normalized grey-tone difference matrix (NGTDM), 48 grey-level dependence zone matrix (GLDZM), and 51 neighborhood grey-level dependence matrix (NGLDM) features. For exact feature definitions, we refer to the document of the Image Biomarker Standardization Initiative^34^.

To eliminate features sensitive to segmentation differences, the intra-class correlation coefficient (ICC) across segmentation approaches was calculated^36^. Features with an ICC below 0.75 were excluded.

All features yielding a Pearson correlation coefficient above 0.9 with volume, SUV_mean_ (mean SUV value of the segmented region) or SUV_max_ (maximum SUV value of the segmented region) were excluded. Next, features were checked for their correlation between each other. If two features were highly correlated (Pearson corr. >0.9), the feature less correlated with volume was kept. After this procedure, a total of 54 features remained. A list of all included features can be found in Supplemental Table 1.

### Classification

A random forest classifier was trained for four classification tasks:

1. Classification of T-stage (low vs. high- stage).
2. Classification of tumor grade (low vs. high grade).
3. Classification of lymph node involvement (yes vs. no).
4. Classification of recurrences.
  - Using clinical values for classification.
  - Using radiomic features for classification.
  - Using clinical values and radiomic features.

T-stage, and tumor grade were transformed to binary values with 0 representing low grade/stage (< 3) and 1 representing a high grade/stage, i.e. 3 or 4.

In tasks 1–3 only radiomic features were used. For task 4, the clinical values T-stage, tumor grading, and lymph node involvement were additionally included.

To get a reliable estimate of classification performance, stratified 10-fold cross-validation was performed. In each fold, the dataset was randomly split in a training and test set, with 90% and 10% of the patients, respectively. To account for class imbalance, the Synthetic Minority Over-Sampling Technique (SMOTE) algorithm^37^ was applied to up-sample the minority class. For comparison, bootstrapping with replacement was performed. Hereby, 95% of the training data was randomly sampled. It was assured that the bootstrapping process was stratified. As results were very comparable between bootstrapping and cross-validation results, the bootstrap results can be found in the Supplemental Material (Supplemental Tables 6–9).

Other classifiers were also tested for their applicability. This included a Support Vector Machine, an AdaBoost classifier, and a number of randomized decision trees. However, as results were comparable across classifiers, we decided to use the random forest in this work. (see Supplemental Fig. 1 for ROC curves of other classifiers).

The hyperparameters of the random forest were optimized by dividing the training set in a stratified manner in training and validation dataset of 80 and 20% size, respectively. For each task, a separate hyperparameter search was performed (see supplemental material for details on hyperparameter search) and the hyperparameters with the overall best performance on the validation dataset were used in this study: The number of estimators was set to 24, gini impurity was used to estimate the split quality, the maximum depth was set to 3, the minimum of samples leading to a split was set to 2. All other hyperparameters were chosen as the default values in scikit-learn and are displayed in the supplemental material.

### Feature reduction

To prevent overfitting, the number of features was reduced to four by using the random forest feature selection using the same random forest hyperparameters as for classification and a maximum number of four features. As for each task different features can be important, we performed the feature selection for each task separately. The feature selection was performed per fold using the actual training set. Please note that we tested also other numbers of selected features, but four features lead to the overall best evaluation metrics. Also, other feature selection methods were tested for their applicability including Principal Component Analysis (PCA), recursive feature elimination (RFE), and the selection of the kBest features. The results across feature selection methods were comparable and we chose the random forest feature selection for final analysis.

### SHAP-values

To increase understanding of the impact of each feature on the classification process, SHapley Additive exPlanations (SHAP) were calculated^38,39^ using the python library SHAP (version 0.44.0). SHAP values assign a feature importance value during classification: Positive SHAP values indicate a contribution to the classification as 1, negative SHAP values indicate a contribution towards 0. By analyzing the SHAP dependence plot of the whole dataset, i.e. comparing feature, corresponding SHAP, and ground truth values, we aim to understand the classifier decision. Hereby, we aim to identify a feature pattern that can separate e.g. high and low-grade tumors. For this purpose, we train the classifier again using (a) all features that were selected in the majority of folds and (b) with each of these features independently. We concentrate on the features selected in the majority of folds as these features should yield a general predictive value for the dataset. In contrast, a feature only selected in one cross-validation fold yields predictive value only for this fold. We compare the classifier decision of one vs. all features to get an impression of feature interaction. I.e. we highlight the patients in whom the combination of multiple features led to a distinct classification compared with using one feature alone. Moreover, SHAP summary plots are displayed for one example fold and each classification task.

### Evaluation of classification performance

The performance of the classifier was evaluated by calculating the accuracy, Area Under the Curve (AUC), positive predictive value (PPV), negative predictive value (NPV), true positive rate (TPR), false positive rate (FPR), F1-score, and Matthews Correlation Coefficient (MCC). Hereby, the accuracy reflects the total accuracy without taking positive or negative samples into account. For imbalanced datasets, the accuracy does not realistically reflect the performance of the classifier as the accuracy can be high if the classifier always decides for the majority class. The AUC reflects the relationship of sensitivity and specificity for different classification thresholds of the model. PPV is the probability that a patient is e.g. classified as having a high tumor grade when the classifier predicts so. The NPV is reversely the probability that a patient is e.g. classified as having a low tumor grade when the classifier predicts so. The FPR is the ratio of false positives to false positives and true negatives. I.e. a FPR equal to 0 means that no false positives were detected. TPR is the ratio of true positives to predict positives in general. A TPR of one indicates that all positive events were correctly identified. The F1-score is the harmonic mean between PPV and TPR. Mathematically, the F1-score is the ratio between two times the number of true positives and the sum of two times the number of true positives and the number of false positives and false negatives. The F1-score can yield values between 1 and 0 with 1 representing perfect PPV and TPR and 0 indicating that at least one of both metrics is 0. The MCC is a correlation coefficient which measures the quality of the classifications. A value of 1 represents a perfect prediction while a value of -1 represents that classes were reversed. A value of 0 represents an average prediction. For all metrics, the mean and standard deviation values across folds were calculated. For AUC also the 95% confidence intervals (CI) were calculated.

In case of a good model accuracy, the calibration of the model was analyzed. To assess the calibration of the classifier, calibration plots plotting the probabilities given by the random forest on the x- and the fraction of patients belonging to the positive class, i.e. having a high T-stage. Additionally, the Brier score measuring the accuracy of probabilistic predictions was calculated. The Brier score is defined as the sum of differences between the expected outcomes and the probabilities given by the random forest divided by the total number of cases.

## Results

### Histopathologic analysis of the study cohort

Patient characteristics are displayed in Table 1. Of 138 patients, 112 (59 (52.7%) men, 53 (47.3%) women) with median age of 63 years (range, 26–87 years) met the inclusion criteria.

**Table 1:** Patient characteristics. Please note that follow-up data was not available for all patients. Hence, only 78 subjects were included in the analysis of recurrence classification.

Patient characteristics	
Sex	59 men
	53 women
Age	63 years (26–87 years)
T- stage	T1: 44
	T2: 37
	T3: 4
	T4: 27
Lymph node involvement	Yes: 34; No: 78
Tumor grade	Grade 1: 2
	Grade 2: 13
	Grade 3: 65
	Grade 4: 28
Recurrence	Yes: 18; No: 60

Histopathologic analysis revealed a tumor category of T1 in 44 patients (39.2%) and T2 in 37 patients (33.0%). Four patients had category T3 (3.6%), and 27 had category T4 (24.1%). 78 patients (69.6%) had no lymph node metastasis (N0), and 34 patients (30.4%) had cervical lymph node involvement. None of the patients investigated showed distant metastasis. 1 patient suffered from tumor grade 1, 13 from grade 2, 65 grade 3, and 28 grade 4.

After a median follow-up of 35 months (standard deviation, 27 months), 15 out of the 78 included patients (19.2%) had experienced tumor recurrence.

### Classification results

#### Classification of T-stage and tumor grade

**Fig. 1:**
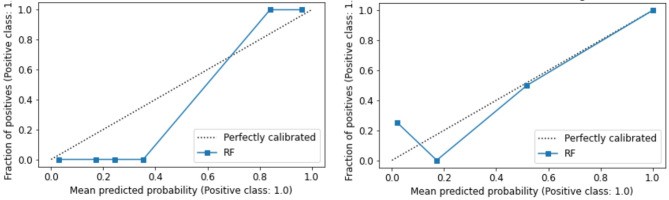
Calibration curves of random forest (RF) model for T-stage classification. As displayed, the RF model follows very well the required line.

The classifier performance to predict low/high T-stage reached a mean accuracy of 85% (standard deviation (std.) 15%) and a mean AUC of 0.82 (std. 0.19, 95% CI [0.68; 0.95]) across folds. The PPV and NPV yielded values of 91% (std. 11%) and 81% (std. 30%). I.e. with a probability of 91% and 81% the tumor classified with a low/high T-stage is a low/high stage tumor, respectively. FPR and TPR yielded mean values of 25% (std. 30%) and 90% (std. 11%). The F1-score resulted in a mean value of 0.89 (std. 0.07) and the MCC in a mean value of 0.66 (std. 0.23). The model is well calibrated as demonstrated by the calibration curves (Fig. 1). The standard deviation of 30% for NPV and TPR indicates that both metrics vary highly across cross-validation folds. This relatively high standard deviation is likely due to the variation in the respective cross-validation training- and test datasets. As also illustrated in the SHAP dependence plots, some high stage tumors share similar features while others don’t. In case of similar feature values in training- and test-set, e.g. the TPR is high. However, if most patients with high-stage tumors and similar feature values are present in the training data, but many patients with high-stage tumors but different feature values are present in the test set, this value is low. As displayed in Fig. 1, the model is well calibrated which is also demonstrated by the mean Brier score of 0.17 (std. 0.05) across folds.

**Fig. 2:**
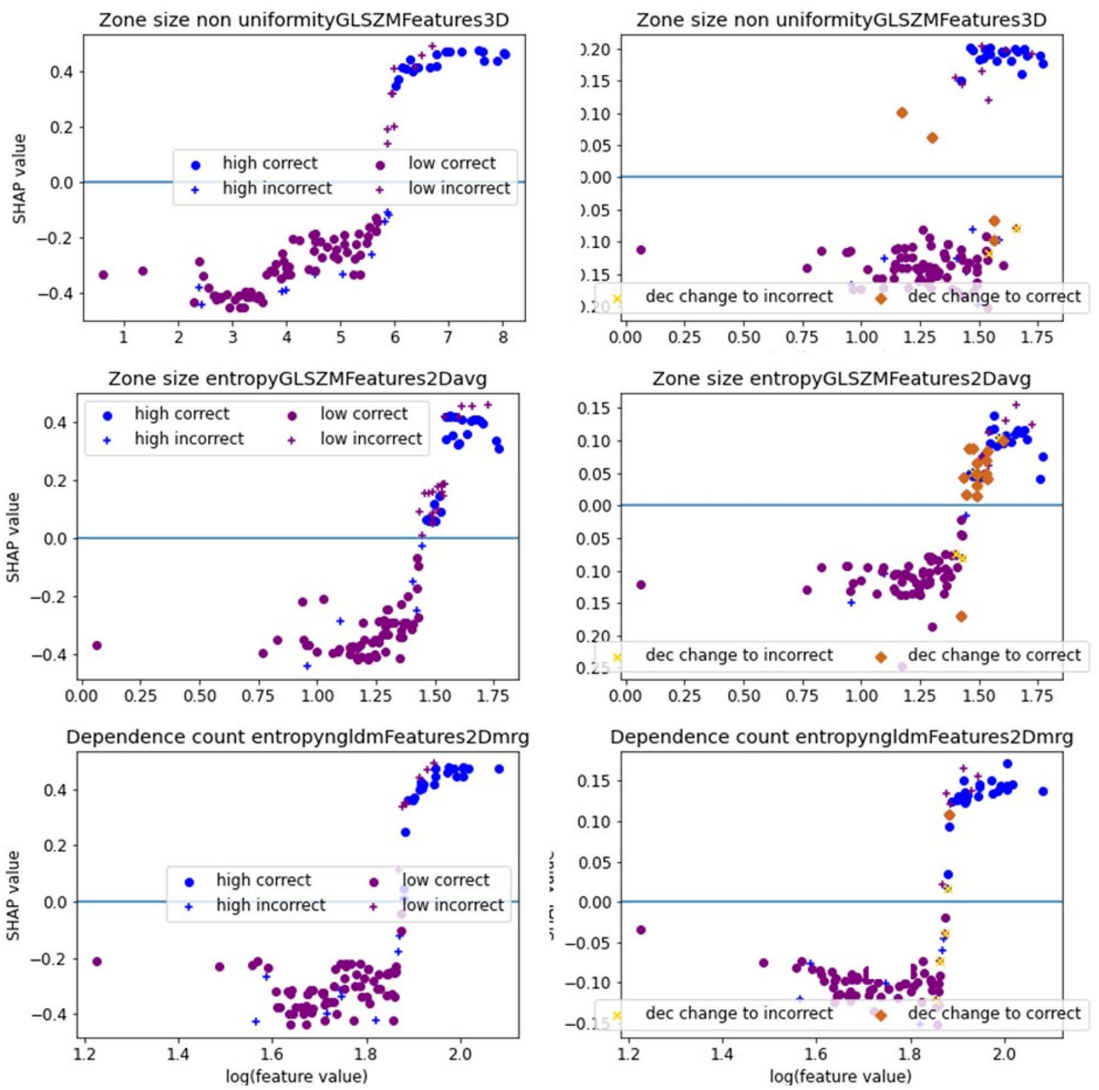
Left: SHAP-dependence plot for features when used alone in T-stage classification. The displayed features are the features most frequently selected across folds: SHAP values > 0 indicate a contribution towards classification to 1 (high-stage), SHAP values < 0 indicate a contribution towards classification to 0 (low-stage). Tumors with high-stage are marked in blue, low-stage in purple, i.e. a blue dot with a negative SHAP value reflects a wrong decision. Ideally, all purple dots would be on one side of the x-axis and all blue dots would be on the other side of the x-axis; In this case, there would be a clear threshold between high and low-stage tumors. Right: SHAP-summary plot when all features were used for classification. Marked in brown/yellow: Classifier decision changed when compared with using the feature alone, i.e. feature interaction had an impact on results especially for the feature zone entropy, for the other two features, the classifier output only changed in a few cases; Please note that SHAP values differ between both columns as both columns belong to different classifiers.

**Fig. 3:**
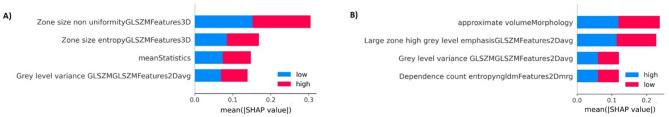
SHAP summary plots for an example fold for classifying tumor stage (left) and tumor grade (right) for one example fold. The displayed features are features selected in this respective fold.

Accuracy metrics for each fold are displayed in Table 2 and feature names for selected features across folds are displayed in Supplemental Table 2. The features selected in most folds were Zone size non-uniformity (GLSZM3D), Dependence count entropy (NGLDM2Dmrg), and Zone size entropy (GLSZM3D) which were selected in 7, 5, and 5 folds, respectively. The corresponding SHAP dependence plots are displayed in Fig. 2. As shown, very high feature values are a clear indication of high-stage tumors. Lower values indicate in more cases a low-stage tumor. In the SHAP dependence plot, the threshold the classifier chose to perform its decision is displayed: I.e. for zone size non-uniformity, all feature values with a logarithm > 6 yield a positive SHAP value and the corresponding tumors are classified as high-stage. However, a few low-stage tumors yield high feature values and are consecutively incorrectly classified as high-stage tumors. In a few cases, these tumors could still be identified as low-stage by training the classifier with all three features. All features yielded similar SHAP values and therefore similar importance. SHAP summary plots of fold 1 (Fig. 3 left) demonstrate that the features Zone size non uniformity (GLSZM3D) has the highest impact on model performance, followed by Zone size entropy (GLSZM3D), mean (Statistics), and Grey level variance (GLSZM2Davg).

**Table 2:** Evaluation metric for each fold for T-stage classification random forest.

Fold number	Accuracy	AUC	PPV	NPV	FPR	TPR
1	0.75	0.75	0.86	0.60	0.25	0.75
2	0.92	0.83	0.90	1.00	0.22	1.00
3	0.64	0.54	0.75	0.33	0.67	0.75
4	0.91	0.83	0.89	1.00	0.33	1.00
5	0.82	0.77	0.88	0.67	0.33	0.88
6	0.91	0.83	0.89	1.00	0.33	1.00
7	0.91	0.83	0.89	1.00	0.33	1.00
8	0.91	0.83	0.89	1.00	0.33	1.00
9	0.91	0.94	1.00	0.75	0.00	0.88
10	0.82	0.77	0.88	0.67	0.33	0.88

For the classification of tumor grade, the classifier achieved a mean accuracy of 55% (std. 13%) and a mean AUC of 0.56 (std. 18%, 95% CI [0.43, 0.68]). The PPV was relatively high with a value of 75% (std. 15%). In contrast, the NPV was very low with 28%. FPR and TPR yielded values of 48% and 56%, respectively. F1-score resulted in mean value of 0.74 (std. 0.07) and MCC in a mean value of 0.26 (std. 0.22) indicating an classification a bit better than average. For tumor grade, the features selected in the most folds were Large zone high grey level emphasis (GLSZM2Davg) and Zone size variance (GLSZM2Dvmrg), which were selected in 4 and 3 folds, respectively. A large number of features were selected in only one fold indicating that there is no feature that has a predictive value for the whole dataset. Accuracy metrics per fold are given in Table 3 and names of selected features are given in Supplemental Table 3. As no feature was selected in the majority of folds, the corresponding SHAP dependence plots are not displayed. The SHAP summary plot of fold 4 (Fig. 3 right) demonstrates that in this fold the features approximate volume (Morphology) and Large zone high grey level emphasis (GLSZM2Davg) yield similar SHAP values, Grey level variance (GLSZM2Davg) Dependence count entropy (NGLDM2Dmrg) have less impact on the model decision.

**Table 3:** Evaluation metrics for the prediction of tumor grade.

Fold number	Accuracy	AUC	PPV	NPV	FPR	TPR
1	0.42	0.44	0.60	0.29	0.50	0.38
2	0.58	0.44	0.64	0.00	1.00	0.88
3	0.64	0.65	0.83	0.40	0.33	0.62
4	0.36	0.35	0.60	0.17	0.67	0.38
5	0.45	0.42	0.67	0.20	0.67	0.50
6	0.55	0.58	0.80	0.33	0.33	0.50
7	0.73	0.81	1.00	0.50	0.00	0.62
8	0.36	0.25	0.57	0.00	1.00	0.50
9	0.64	0.65	0.83	0.40	0.33	0.62
10	0.73	0.81	1.00	0.50	0.00	0.62

**Fig. 4:**
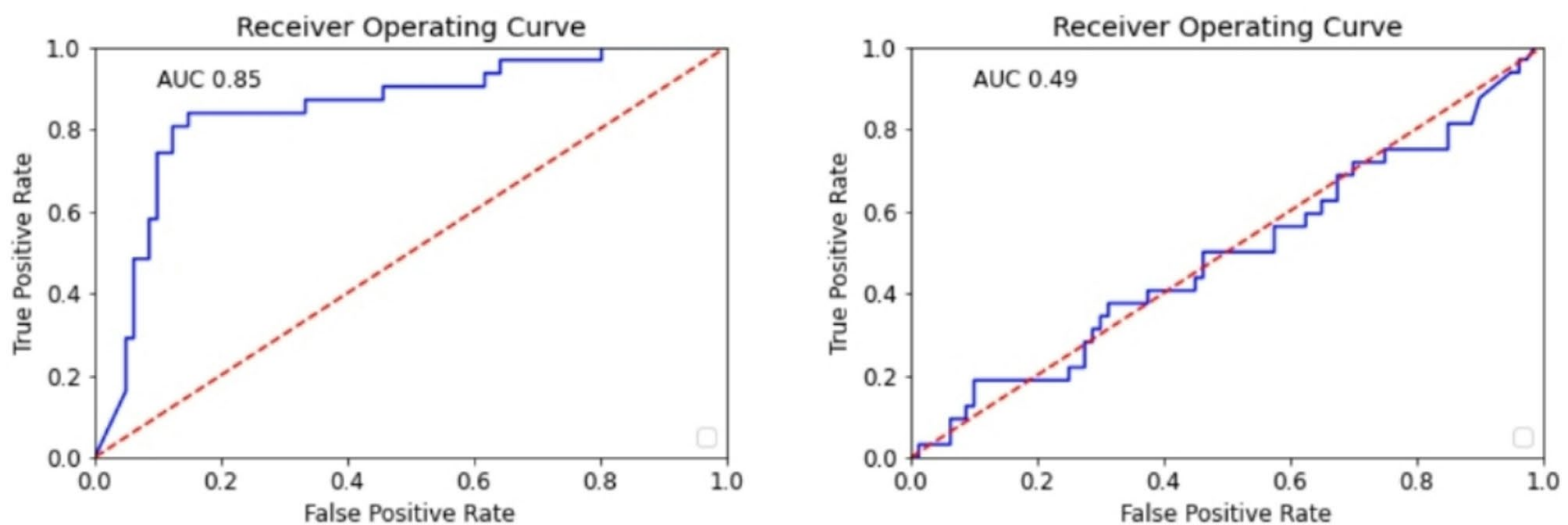
ROC curve for the classification of T-stage (left) and tumor grade (right).

ROC curves for both classification tasks are displayed in Fig. 4.

#### Classification of lymph node involvement and recurrence

**Fig. 5:**
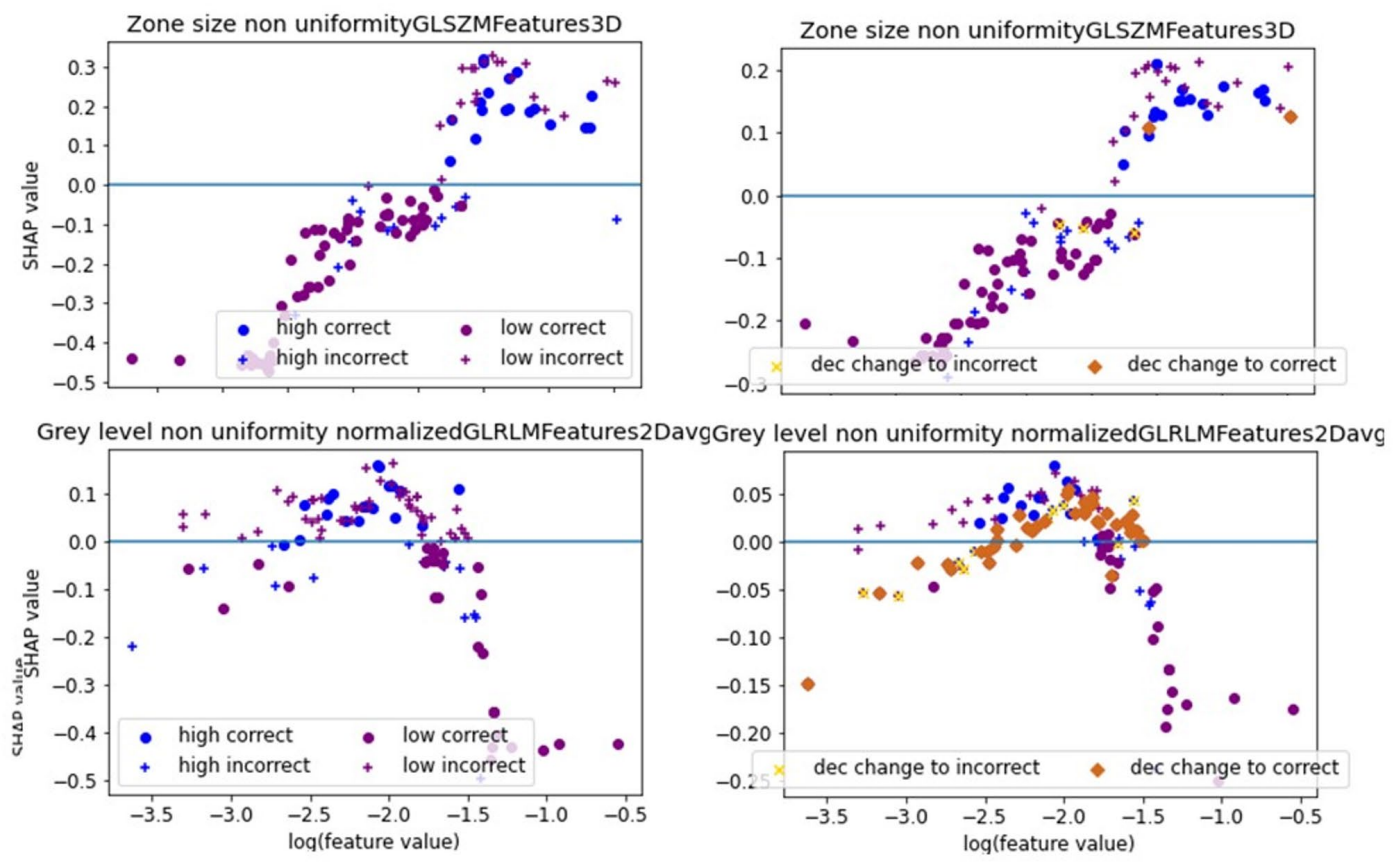
SHAP dependence plots for lymph node involvement. For a more explicit explication of the plots, we refer to Fig. 2. No clear threshold between patients with lymph node involvement (blue) and without lymph node involvement (purple) can be observed: Ideally, all blue and all purple dots would be on one side of the x-axis.

When predicting lymph node involvement, the classifier reached a mean accuracy of 67% (std. 9%) and a mean AUC of 0.64 (std. 0.11, 95% CI [0.56; 0.71]). A PPV of 82% (std. 10%) indicated that in 82% of the cases the classifier indicates presence of lymph node metastasis, the patient suffered from tumor spread to the lymph nodes. In contrast, the NPV of 53% (std. 25%) was low. FPR was also low with 42% (std. 29%), while TPR yielded 70% (std. 19%). Also here, a standard deviation of 25% for NPV and of 29% for FPW indicate that both metrics vary highly across cross-validation folds. The reason for this effect is also here in the fact that no feature can predict lymph node involvement accurately. A tumor with and a tumor without lymph node involvement can yield very similar feature values. Depending on the distribution of these values in training- and test-set, the evaluation metrics can vary highly. The accuracy metrics per fold are listed in Table 4. The features Grey level non-uniformity normalized (GLRLM2Davg) and Zone size non-uniformity were selected in 4 folds (Supplemental Table 4). The SHAP dependence plot of both features shows that the classifier selects a threshold for both features with whom the classifier is trying to separate both classes. E.g. for the feature Zone size non-uniformity: the classifier identifies patients with a logarithmic of the feature value above 5.5 as patients with lymph node involvement. As can be seen in the dependence plot, this threshold is not capable of dividing the patients correctly in the right classes (Fig. 5). However, all patients with very low feature values are indeed patients with no lymph node involvement. SHAP summary plots of an example fold (Fig. 7) indicate that the feature approximate volume (Morphology) has the highest impact on model performance, followed by Grey level non uniformity normalized (GLDZM2Dmrg), Dependence count entropy (NGLDM2Dmrg), and High dependence high grey level emphasis. (NGLDMD3Dmrg).

**Table 4:** Evaluation metrics for different folds for the prediction of lymph node involvement.

Fold number	Accuracy	AUC	PPV	NPV	FPR	TPR
1	0.58	0.63	0.8	0.43	0.25	0.5
2	0.67	0.63	0.75	0.50	0.50	0.75
3	0.55	0.65	1.0	0.44	0.0	0.28
4	0.82	0.75	0.78	1.0	0.50	1.0
5	0.64	0.75	1.00	0.43	0.00	0.5
6	0.55	0.48	0.72	0.25	0.67	0.63
7	0.82	0.66	0.80	1.0	0.67	1.0
8	0.73	0.71	0.86	0.50	0.33	0.75
9	0.64	0.54	0.75	0.33	0.67	0.75
10	0.72	0.60	0.78	0.50	0.67	0.88

**Fig. 6:**
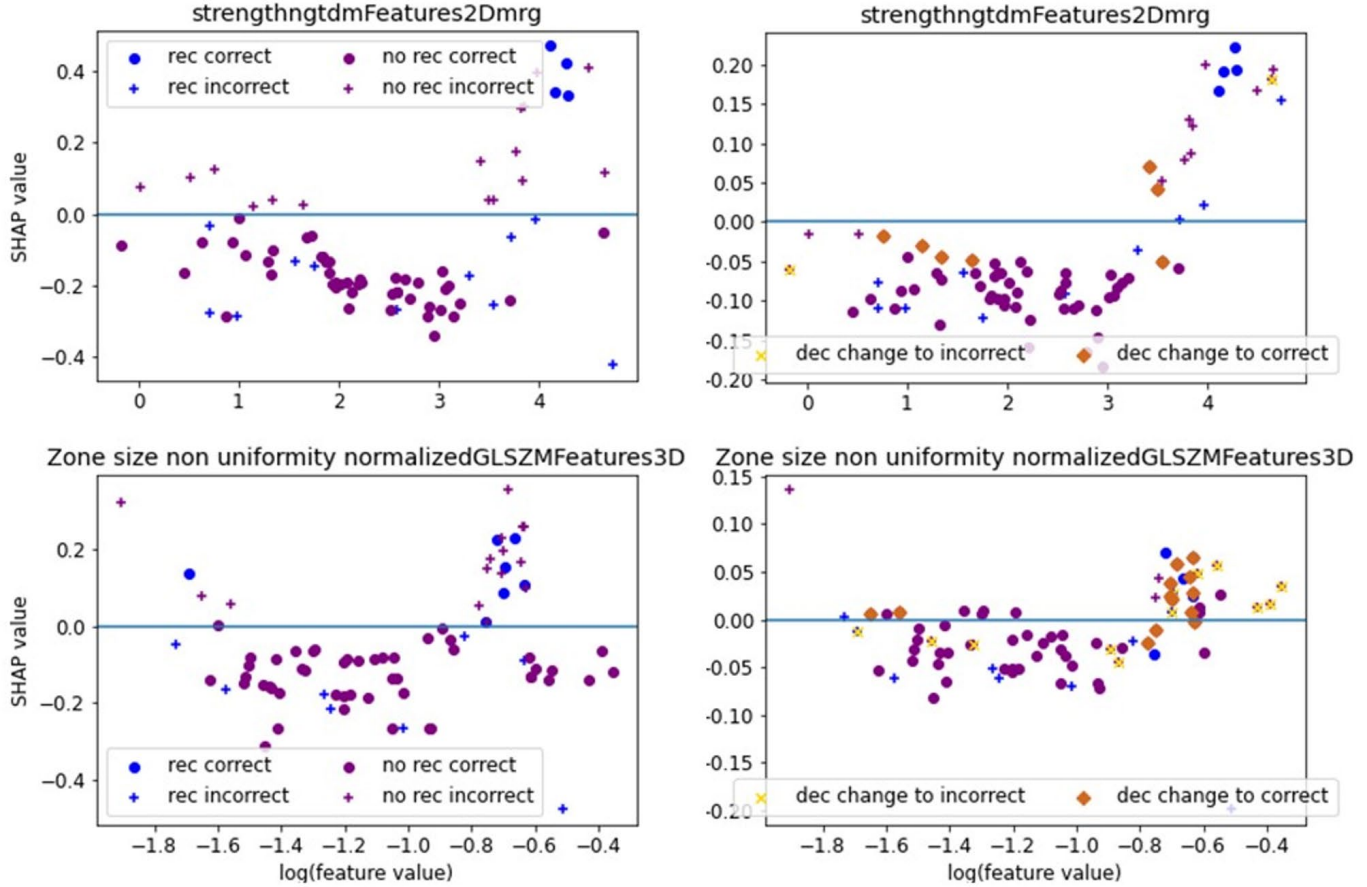
SHAP dependence plot for recurrence classification. For a deeper explanation, please check Fig. 2.

**Fig. 7:**
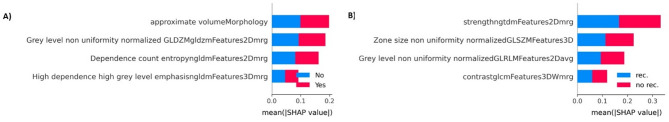
SHAP summary plot for lymph node involvement (left) and tumor recurrence (right).

When predicting tumor recurrence by using radiomic features only, a mean accuracy of 70% (std. 20%), a mean AUC of 0.63 (std. 0.28, 95% CI [0.43; 0.82]), a PPV of 85% (std. 13%), and an NPV of 31% (std. 31%) were observed (Supplemental Table 8). FPR and TPR yielded values of 50% (std. 44%) and 76% (std. 17%), respectively. The mean F1-score resulted in a value of 0.61 (std. 0.08) and the MCC resulted in a mean value of -0.14 (std. 0.16). The accuracy metrics per fold are listed in Table 5. The features strength (NGTMD2DMRG) and Zone size non-uniformity normalized (GLSZM3D) were selected in most folds – namely in 10 and 6 folds (Supplemental Table 5), respectively. When analyzing the SHAP dependence plots (Fig. 6), no clear threshold between feature values of patients with and without recurrence is visible. The classifier identifies a feature pattern that cannot separate the two cases correctly. The combination of both features led to more correct classifications than when using one feature alone. SHAP summary plots of an example fold (Fig. 7) indicate that the feature Strength (NGTDM2Dmrg), and Zone size non uniformity normalized (GLSZM3D) have the highest impact on model performance in this fold, followed by Grey level non uniformity normalized (GLRLM2Davg) and contrast (GLCM3DWmrg).

**Table 5:** Evaluation metrics for different folds for the prediction of tumor recurrence.

Fold number	Accuracy	AUC	PPV	NPV	FPR	TPR
1	0.88	0.93	1.00	0.50	0.00	0.86
2	0.88	0.93	1.00	0.50	0.00	0.86
3	0.75	0.43	0.86	0.00	1.00	0.86
4	0.62	0.42	0.71	0.00	1.00	0.83
5	1.00	1.00	1.00	1.00	0.00	1.00
6	0.62	0.58	0.80	0.33	0.50	0.67
7	0.50	0.33	0.67	0.00	1.00	0.67
8	0.62	0.58	0.80	0.33	0.50	0.67
9	0.86	0.92	1.00	0.50	0.00	0.83
10	0.29	0.17	0.67	0.00	1.00	0.33

When using radiomic and clinical features, the accuracy dropped slightly to an accuracy of 66% (std. 14%) and an AUC of 0.55 (std. 0.21, 95% CI [0.40; 0.70]). PPV and NPV reached values of 83% and 25%, respectively. FPR and TPR yielded values of 65% and 74%. The model including only clinical features resulted in a mean accuracy and AUC values of 0.47 (std. 0.21, 95% CI [0.32; 0.62] ) and 0.46 (std. 0.28). PPV, NPV, FPR, and NPR yielded values of 78.7% (std. 21%), 17% (std. 19%), 55% (std. 47%), and 47% (std. 19%), respectively.

In this case, the radiomic model performed better than clinical features. However, as demonstrated by the SHAP dependence plots, there was no feature that showed a predictive value for the whole dataset. In contrast, different features were selected in each cross-validation fold. Therefore, radiomic features seem to have no value for recurrence identification.

**Fig. 8:**
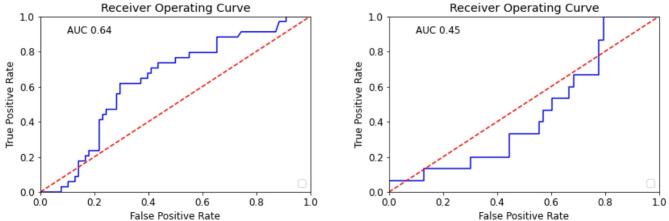
ROC curves for the classification of lymph node involvement (left) and recurrence (right).

ROC curves for both tasks and the pure radiomics models are displayed in Fig. 8.

## Discussion

In this prospective study, we explored the value of radiomic features in treatment-naïve patients with newly diagnosed OSCC prior to tumor resection and neck dissection. We demonstrate that radiomic features can separate advanced and early T-stage of primary OSCC with good accuracy. However, they fail to classify tumor grade, lymph node involvement, and recurrence.

A study by Martens et al. showed that in patients with treatment-naive head and neck squamous cell carcinomas the combination of clinical variables with radiomic features was most accurate for predication of recurrence (HPV-status, first order [^18^F]FDG PET/CT parameters as well as complementary radiomic features) and metabolic-active tumor volume for prediction of distant metastasis^40^. However, the precise value of radiomic features extracted from PET images only is not clear. Consequently, we focused on using image data only. Regarding the radiomic features used in our study for classification, we applied strict criteria to minimize potential correlations to known confounding factors such as volume and to include only features stable across segmentations. However, as a consequence, features that do have a predictive value might be eliminated. Our results demonstrate that with our strict approach the performance for the classification of tumor grade, lymph node involvement, and recurrence was low. However, these negative results are in line with studies in other tumor entities demonstrating a rather low performance of PET radiomics only. E.g., in a recent study by Collarino and colleagues investigating the predictive value of radiomics extracted from PET scans of patients suffering from locally advanced cervical cancer, radiomic features failed to classify overall survival and tumor recurrence^25^. In this vein, Eertink et al. demonstrated that conventional PET parameters in combination with dissemination features outperformed radiomic features for classifying progression in DLBCL patients^24^.

For T-stage classification, high Zone size non-uniformity (GLSZM3D), Dependence count entropy (NGLDM2Dmrg), and Zone size entropy (GLSZM3D) values indicated tumors with an advanced stage. Hereby, Zone size non-uniformity describes the distribution of grey-level zone sizes. A zone represents a connected area of the same discretized grey level. If the value is low, all grey levels have similar zone sizes. Zone size entropy represents how zones of the same size and grey level are distributed. Dependence count entropy is calculated from the NGLDM feature group which aims to represent texture coarseness. Dependence count entropy is low when the tumor is rather homogeneous while it is high for a homogeneous tumor. Previous works used radiomic features extracted from PET scans of OSCC patients to classify overall and progression-free survival^21^ and found also the feature Zone size entropy (GLSZM) to yield a high predictive value. Future work should investigate which tumor characteristics this feature exactly represents.

This study has several strengths and limitations. Noteworthy, this is the first prospective evaluation of the role of radiomic features in a homogenous cohort of newly diagnosed, treatment-naive patients, all undergoing a uniform treatment (surgery) after imaging. Another strength is that a majority vote approach was used to segment the primary tumor, which is known to lead to stable and more reproducible results^32^. To allow for the reproducibility of our results, all features were extracted using software^33^ that complies with the guidelines of the Imaging Biomarker Standardization Initiative^34^. By performing cross-validation and comparing selected features across folds, we demonstrated for almost all tasks (except of T-staging) that no feature had a predictive value.

However our study suffers from various limitations. First, data was only collected from a single institution and not tested in an external group. To overcome the limitation that only a limited number of patients were included in this study, we analyzed the SHAP dependence plots and demonstrated that no feature yielded significant differences between e.g. recurrence and no recurrence. These results indicate that the failure of the radiomic model is not due to the data size but rather due to the low prognostic power of the features. These results need to be confirmed in a larger patient cohort.

Further we used SHAP dependence plots to explain the classifier’s decisions. However, the SHAP dependence plots do not give a clear understanding of feature interactions. Future work should investigate the impact of feature interaction on classification results.

Another limitation is that we only used PET radiomics features as imaging modality features. In a recent study by Nikkuni et al. a machine learning model with preoperative PET radiomics features was used to diagnose the histological grade with an AUC up to 0.84. However no other tumor characteristics were examined, and results were also not tested in an external group^40–43^.

Further studies discuss the additional value for CT-and MRI radiomics features in head and neck squamous cell cancers and suggest that the combination of radiomic features from different modalities, i.e. CT, MRI and PET need to be investigated in future studies for potential improvement of prediction accuracy^44^. Moreover, the potential clinical impact of PET radiomics and our model needs to be further evaluated.

In summary, this study demonstrates that there might be some potential for using radiomic features for classifying the T-stage of OSCC patients with PET radiomic features. However, our investigations need to be confirmed in a larger patient cohort where also data from different hospitals and PET scanners are included.

## Conclusion

Radiomic features extracted from PET scans of OSCC patients failed to accurately predict tumor grade, lymph node involvement, and risk of recurrence. In contrary, PET radiomic features yielded a good accuracy when classifying tumor stage. However, the results for T-stage classification need to be validated in a multi-center setting.

## Electronic supplementary material

Below is the link to the electronic supplementary material.

Supplementary Material 1

## Data Availability

The dataset used and analyzed during the current study are available anonymized from the corresponding author on reasonable request.
