# Supplementary material for: Value of PET radiomic features for diagnosis and reccurence prediction of newly diagnosed oral squamous cell carcinoma
Source: Sci Rep. 2025 May 20;15:17475. doi: 10.1038/s41598-025-02305-3 (PMC12092716; doi:10.1038/s41598-025-02305-3)
Supplement: Supplementary file 1 — Supplementary Material 1 [file 41598_2025_2305_MOESM1_ESM.docx]

# SUPPLEMENTAL MATERIAL

| **Feature name (group)** |  |  |
| --- | --- | --- |
| Approximate volume (Morphology) | Asphericity (Morphology) | Major axis length (Morphology) |
| Mean (Statistics) | Mean absolute deviation (Statistics) | Median absolute deviation (Intensity histogram) |
| Difference variance (GLCM2Davg) | Contrast (GLCM3DWmrg) | Dissimilarity (GLCM3DWmrg) |
| Short run high grey level emphasis (GLRLM2Davg) | Long run high grey level emphasis (GLRLM2Davg) | Grey level non uniformity normalized (GLRLM2Davg) |
| Grey level non uniformity normalized (GLRLM3Dmrg) | Run length non uniformity normalized (GLRLM3Dmrg) | Grey level variance (GLRLM3Dmrg) |
| Large zone high grey level emphasis (GLSZM2Davg) | Grey level variance (GLSZM2Davg) | Zone size entropy (GLSZM2Davg) |
| Grey level variance (GLSZM2Dvmrg) | Zone size variance (GLSZM2Dvmrg) | Zone size non uniformity (GLSZM3D) |
| Grey level variance (GLSZM3D) | Zone size entropy (GLSZM3D) | Complexity (NGTDM2Dmrg) |
| Strength (NGTDM2Dmrg) | Grey level non uniformity normalized (GLDZM2Dmrg) | Grey level variance (GLDZM3D) |
| High dependence high grey level emphasis (NGLDM2Davg) | Low dependence emphasis (NGLDM2Dmrg) | Dependence count entropy (NGLDM2Dmrg) |
| High dependence high grey level emphasis (NGLDM3Dmrg) | Dependence count non uniformity normalized (NGLDM3Dmrg) | Grey level variance (NGLDM3Dmrg) |
| Dependence count entropy (NGLDM3Dmrg) | Dependence Count Energy (NGLDM3Dmrg) |  |

Supplemental Table 1: Feature names of remaining features; Features instable over different segmentation methods, highly correlated with volume, SUV_mean_, and SUV_max_ were eliminated

***Details on hyperparameter search:***

In brackets are the values included in the hyperparameter search:

N_estimators = {8,16,24,48,68,96}

Criterion={’gini’, ‘entropy’}; Default: ‘gini’ (also chosen by search)

Max_depth= {2, 3, 5, 10}

Min_samples_split: {2, 5, 10}

Min_samples_leaf=1, min_weight_fraction_leaf=0.0, max_features='sqrt',

max_leaf_nodes=None, min_impurity_decrease=0.0, bootstrap=True, oob_score=False

n_jobs=None, random_state=None, verbose=0, warm_start=False, class_weight=None

ccp_alpha=0.0, max_samples=None, monotonic_cst=None


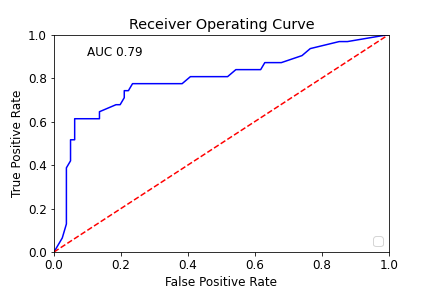

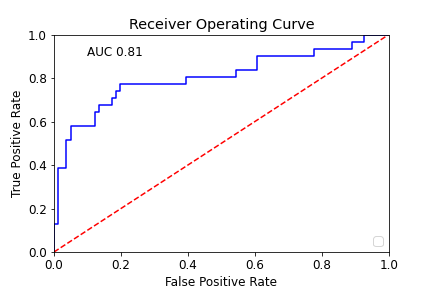


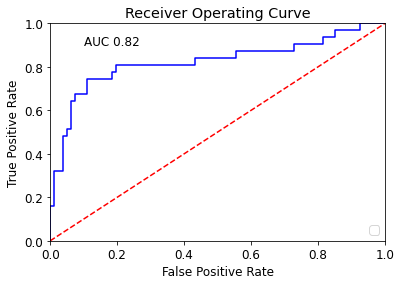


Supplemental Fig. 1: AUC curves for T-stage classification for SVM (upper left), decision trees (upper right), and AdaBoost (lower left). As displayed, results are similar across classifiers.

| **Feature name (Feature group)** | **Nr. of folds in which feature was selected** |
| --- | --- |
| Mean (statistics) | 2 |
| Grey level variance (GLSZM2Davg) | 1 |
| Zone size non uniformity  (GLSZM3D) | 7 |
| High dependence high grey level emphasis (ngldm2Davg) | 1 |
| Grey level non uniformity normalized (GLDZM2Dmrg) | 4 |
| Dependence count entropy (NGLDM2Dmrg) | 5 |
| Grey level variance (GLSZM3D) | 1 |
| Long run high grey level emphasis (GLRLM2Davg) | 4 |
| Grey level non uniformity normalized (GLRLM2Davg) | 1 |
| Grey level non uniformity normalized (GLRLM3Dmrg) | 1 |
| Major axis length (Morphology) | 2 |
| Zone size entropy (GLSZM3D) | 5 |
| Grey level variance (GLRLM3Dmrg) | 1 |
| Median absolut deviation (Intensity histogram) | 2 |
| Zone size entropy (GLSZM2Davg) | 2 |
| Short run high grey level emphasis (GLRLM2Davg) | 3 |

Supplemental Table 2: Selected feature for T-stage classification

| **Feature name (Feature group)** | **Nr. of folds in which feature was selected** |
| --- | --- |
| Large zone high grey level emphasis (GLSZM2Davg) | 4 |
| Strength (NGTDM2Dmrg) | 2 |
| High dependence high grey level emphasis (NGLDM2Davg) | 2 |
| High dependence high grey level emphasis (NGLDM3Dmrg) | 1 |
| Dependence count entropy (NGLDM2Dmrg) | 2 |
| Asphericity (Morphology) | 2 |
| Zone size entropy (GLSZM2Davg) | 1 |
| Grey level variance (GLDZM3D) | 2 |
| Mean absolut deviation (Statistics) | 2 |
| Approximate volume (Morphology) | 1 |
| Major axis length (Morphology) | 2 |
| Dissimilarity (GLCM3DWmrg) | 2 |
| Zone size variance (GLSZM2Dvmrg) | 3 |
| Grey level non uniformity normalized (GLDZM2Dmrg) | 2 |
| Difference variance (GLCM2Davg) | 2 |
| Long run high grey level emphasis (GLRLM2Davg) | 1 |
| Low dependence emphasis (NGLDM2Dmrg) | 1 |
| Mean (Statistics) | 1 |
| Run length non uniformity normalized (GLRLM3Dmrg) | 1 |
| Low dependence emphasis (NGLDM2Dmrg) | 1 |
| Complexity (NGLDM2Dmrg) | 1 |
| Grey level non uniformity normalized (GLRLM3Dmrg) | 1 |
| Grey level variance (GLSZM2Davg) | 1 |
| Zone size entropy (GLSZM3D) | 2 |

Supplemental Table 3: Selected features to predict tumor grade

| **Feature name (Feature group)** | **Nr. of folds in which feature was selected** |
| --- | --- |
| Zone size entropy (GLSZM3D) | 2 |
| Grey level variance (GLRLM3D) | 1 |
| Long run high grey level emphasis (GLRLM2Davg) | 2 |
| Grey level non uniformity normalized(GLRLM2Davg) | 4 |
| Grey level variance (GLSZM2Dvmrg) | 1 |
| Grey level variance (GLDZM3D) | 2 |
| Mean absolut deviation (Statistics) | 1 |
| Approximate volume (Morphology) | 2 |
| Major axis length (Morphology) | 3 |
| Dissimilarity (GLCM3DWmrg) | 2 |
| Zone size non uniformity (GLSZM2Dvmrg) | 4 |
| Difference variance (GLCM2Davg) | 2 |
| Low dependence emphasis (NGLDM2Dmrg) | 1 |
| Dependence count non uniformity normalized (NGLDM3Dmrg) | 3 |
| Run length non uniformity normalized (GLRLM3Dmrg) | 1 |
| Grey level non uniformity normalized (GLDZM2Dmrg) | 2 |
| Contrast (GLCM3DWmrg) | 2 |
| High dependence high grey level emphasis (NGLDM3Dmrg) | 3 |
| Dependence count entropy (NGLDM2Dmrg) | 1 |
| Short run high grey level emphasis (GLRLM2Davg) | 2 |
| Complexity (NGTDM2Dmrg) | 1 |
| Strength (NGTDM2Dmrg) | 1 |
| Grey level non uniformity normalized (GLDZM2Dmrg) | 1 |

Supplemental Table 4: Selected feature names for predicting lymph node involvement

| **Feature name (Feature group)** | **Nr. of folds in which feature was selected** |
| --- | --- |
| Grey level non uniformity normalized (GLRLM3Dmrg) | 4 |
| Strength (ngtdm2Dmrg) | 10 |
| Grey level non uniformity normalized (GLDZM2Dmrg) | 2 |
| High dependence high grey level emphasis (ngldm3Dmrg) | 2 |
| Approximate volume (Morphology) | 1 |
| Grey level non uniformity normalized (GLRLM2Davg) | 3 |
| Zone size non uniformity normalized (GLSZM3D) | 6 |
| Short run high grey level emphasis (GLRLM2Davg) | 1 |
| High dependence high grey level emphasis (NGLDM3Dmrg) | 1 |
| Mean (statistics) | 1 |
| Major axis length (Morphology) | 1 |
| Difference variance (GLCM2Davg) | 1 |
| Contrast (GLCM3DWmrg) | 1 |
| Dependence count entropy (NGLDM3Dmrg) | 2 |
| Zone size variance (GLSZM2Dvmrg) | 2 |
| Zone size non uniformity (GLSZM3D) | 2 |

Supplemental Table 5: Selected feature for recurrence prediction

| Classification task | Cross-validation | Bootstrapping |
| --- | --- | --- |
| T-stage | 85 % (std. 15%) | 84 % (std. 14 %) |
| Tumor grade | 55 % (std. 13%) | 54 % (std. 12 %) |
| Lymph node involvement | 67 % (std. 9%) | 66 % (std. 10%) |
| Recurrence | 70 % (std. 20 %) | 68 % (std. 21%) |

Supplemental Table 6: Comparison bootstrap and cross-validation mean and std. of accuracy

| Classification task | Cross-validation | Bootstrapping |
| --- | --- | --- |
| T-stage | 0.82 (std. 0.19) | 0.81 (std. 0.15) |
| Tumor grade | 0.56 (std. 0.18) | 0.54 (std. 0.19) |
| Lymph node involvement | 0.64 (std. 0.11) | 0.64 (std. 0.10) |
| Recurrence | 0.63 (std. 0.28) | 0.62 (std. 0.29) |

Supplemental Table 7: Comparison bootstrap and cross-validation mean and std. of AUC

| Classification task | Cross-validation | Bootstrapping |
| --- | --- | --- |
| T-stage | 91%(std.11%)/81%(std.30%) | 92%(std.11%)/80%(std.29%) |
| Tumor grade | 75%(std.15%)/28%(std.21%) | 76%(std.14%)/27%(std.20%) |
| Lymph node involvement | 82%(std.10%)/53%(std.25%) | 81%(std.11%)/54%(std.23%) |
| Recurrence | 85%(std. 13%) / 31%(std.31%) | 84%(std.14%)/32%(std.29%) |

Supplemental Table 8: Comparison bootstrap and cross-validation mean and std. of PPV/NPV

| Classification task | Cross-validation | Bootstrapping |
| --- | --- | --- |
| T-stage | 25%(std.30%)/90%(std.11%) | 23%(std.31%)/89%(std.10%) |
| Tumor grade | 48%(std.17%)/56%(std.23%) | 49%(std.18%)/55%(std.22%) |
| Lymph node involvement | 50%(std.44%)/76%(std.17%) | 51%(std.42%)/74%(std.16%) |
| Recurrence | 55%(std. 47%) / 47%(std.19%) | 54%(std.46%)/48%(std.21%) |

Supplemental Table 9: Comparison bootstrap and cross-validation mean and std. of FPR/TPR
